# Anti-Müllerian Hormone Levels Across Phenotypes in Polycystic Ovary Syndrome: A Systematic Review and Meta-analysis

**DOI:** 10.1210/jendso/bvaf199

**Published:** 2025-12-16

**Authors:** Patrícia Jorge Schwenck-Carvalho, Giovana De Nardo Maffazioli, Ricardo Santos Simões, Renata Ferri Macchione, Sebastião Freitas de Medeiros, José Maria Soares Júnior, Edmund Chada Baracat, Gustavo Arantes Rosa Maciel

**Affiliations:** Discipline of Gynecology, Hospital das Clinicas HCFMUSP, Faculdade de Medicina, Universidade de Sao Paulo, Sao Paulo, SP 05403-000, Brazil; Discipline of Gynecology, Hospital das Clinicas HCFMUSP, Faculdade de Medicina, Universidade de Sao Paulo, Sao Paulo, SP 05403-000, Brazil; Discipline of Gynecology, Hospital das Clinicas HCFMUSP, Faculdade de Medicina, Universidade de Sao Paulo, Sao Paulo, SP 05403-000, Brazil; Discipline of Gynecology, Hospital das Clinicas HCFMUSP, Faculdade de Medicina, Universidade de Sao Paulo, Sao Paulo, SP 05403-000, Brazil; Department of Gynecology and Obstetrics, Medical School, Federal University of Mato Grosso, Cuiaba, MT 78055-728, Brazil; Discipline of Gynecology, Hospital das Clinicas HCFMUSP, Faculdade de Medicina, Universidade de Sao Paulo, Sao Paulo, SP 05403-000, Brazil; Discipline of Gynecology, Hospital das Clinicas HCFMUSP, Faculdade de Medicina, Universidade de Sao Paulo, Sao Paulo, SP 05403-000, Brazil; Discipline of Gynecology, Hospital das Clinicas HCFMUSP, Faculdade de Medicina, Universidade de Sao Paulo, Sao Paulo, SP 05403-000, Brazil

**Keywords:** polycystic ovary syndrome, anti-Müllerian hormone, hyperandrogenism, oligo-anovulation, polycystic ovarian morphology, meta-analysis

## Abstract

**Context:**

Anti-Müllerian hormone (AMH) concentrations are frequently elevated in women with polycystic ovary syndrome (PCOS). However, clinical application is limited by the absence of a standardized cutoff, lack of assay harmonization, and variability across PCOS phenotypes.

**Objective:**

To compare AMH levels across the 4 PCOS phenotypes (A, B, C, D) defined by the Rotterdam criteria.

**Data sources:**

PubMed, Embase, ScienceDirect, and Web of Science were searched for studies published between January 2009 and July 2024. Reference lists of included studies were also screened.

**Study selection:**

Eligible studies included women with PCOS diagnosed by Rotterdam criteria and reported AMH levels by phenotype. Of 684 citations, 49 studies (15 535 participants) met inclusion criteria.

**Data extraction:**

Two reviewers independently extracted study characteristics, AMH values, assay type, age, and body mass index (BMI). AMH levels were converted to Beckman Coulter Gen II units. Risk of bias was assessed using ROBINS-E.

**Data synthesis:**

Random-effects meta-analyses showed highest AMH levels in phenotype A (11.49 ng/mL; standardized mean difference 3.06), followed by D (8.97), C (7.98), and B (6.25; standardized mean difference 1.36). Age and BMI were comparable. Heterogeneity was high (*I*^2^ ≈ 98%). Meta-regression adjusted for phenotype, age, BMI, and region confirmed the AMH hierarchy (A > D ≈ C > B) and identified geographic region as a significant contributor to heterogeneity.

**Conclusion:**

AMH levels differ markedly across PCOS phenotypes, with polycystic ovarian morphology exerting the greatest influence, followed by oligoanovulation and hyperandrogenism. Findings support the use of AMH in phenotype differentiation and emphasize the need for assay standardization and population-specific interpretation.

Polycystic ovary syndrome (PCOS) is a complex pathological condition characterized by hyperandrogenism, ovulatory dysfunction, and polycystic ovaries. It affects women of reproductive age, with life-long impacts from adolescence to postmenopause [1]. It is a disorder, with an incidence varying from 4% to 17% of women in their reproductive years [2].

Different consensus of diagnostic criteria for PCOS were designed but the most widely accepted is the Rotterdam criteria (2003), which was updated by the latest international PCOS guidelines in 2023 [1]. According to the combination of Rotterdam criteria used for the diagnosis of PCOS, the syndrome can be classified into phenotypic presentations: phenotype A (PCOS-A)—hyperandrogenism (HA), oligo-amenorrhea, and polycystic ovarian morphology (PCOM) on ultrasound; phenotype B (PCOS-B)—HA and oligo-amenorrhea; phenotype C (PCOS-C)—HA and PCOM; phenotype D (PCOS-D)—oligo-amenorrhea and PCOM [3].

Despite their widespread acceptance, the Rotterdam diagnostic criteria continue to prompt discussion: the assessment of clinical and laboratory parameters of hyperandrogenism and hyperandrogenemia, the definition of oligo-amenorrhea throughout the reproductive age, the criteria to define the polycystic appearance of the ovaries on the ultrasound, the role of anti-Müllerian hormone (AMH) in the diagnosis and phenotypes, among others, make its management a clinical challenge [4]. There is a trend in the search for objective diagnostic criteria that encourages research into biomarkers in PCOS.

A promising diagnostic parameter is AMH, a dimeric glycoprotein that plays an important role in sexual differentiation and regulation of folliculogenesis. It is named for its ability to inhibit the development of the Müllerian ducts in male fetuses [5]. AMH regulates ovarian folliculogenesis by inhibiting the recruitment of primordial follicles [6]. There are several possible uses for the AMH assessment: evaluation of the ovarian reserve, prediction of controlled ovarian stimulation, prediction of the natural age of menopause, assessment of the ovarian function, differentiation of some disorders of sex development, and tumor marker [7]. It is known that women with PCOS present elevated levels of AMH, correlating with testosterone levels, free androgen index, LH, average ovarian volume, the number of follicles on transvaginal ultrasound, and also with ovulatory dysfunction, including oligomenorrhea and amenorrhea [5]. Although some authors have proposed AMH cutoff levels capable of distinguishing PCOS from non-PCOS patients with reasonable sensitivity and specificity [8], current international guidelines recommend AMH only as an alternative method for identifying PCOM [1]. However, the influence of each Rotterdam phenotype on AMH levels may impact its utility as a diagnostic indicator for PCOS [7].

## Objectives

This systematic review aims to comprehensively evaluate and compare the mean levels of AMH among the different phenotypes (A, B, C, and D) of PCOS. Additionally, we aim to explore potential factors that may influence AMH levels within each phenotype, assess heterogeneity across studies, and conduct a meta-analysis to provide summary estimates of AMH levels in each PCOS phenotype.

## Materials and Methods

### Eligibility Criteria, Information Sources, Search Strategy

The present research has been registered on the PROSPERO platform under the number CRD42023444193 and follows the Preferred Reporting Items for Systematic Reviews and Meta-analysis (PRISMA) statement [9]. This systematic review includes studies that meet the following criteria: women diagnosed with PCOS based on the Rotterdam criteria; studies that specifically report AMH levels categorized into PCOS phenotypes A, B, C, and D; studies published in English, Spanish, Portuguese, or French; both prospective and retrospective studies; and all types of study designs (randomized controlled trials, cohort studies, case-control studies, cross-sectional studies) as long as they provide the necessary data. The presence of a healthy control group was not required, and most included studies did not provide such data.

The exclusion criteria involve studies that do not include populations diagnosed with PCOS according to the Rotterdam criteria. These studies do not provide specific data on AMH levels, age, and body mass index (BMI), studies that do not distinguish between different phenotypes of PCOS according to the Rotterdam criteria, animal studies, reviews, case reports, opinion articles, letters to the editor, and other nonoriginal research.

Two independent reviewers performed a comprehensive search of PubMed, Embase, Science Direct, and Web of Science databases for studies published up to July 2024. The following search terms were applied: “PCOS” or “Polycystic Ovary Syndrome,” “AMH” or “Anti-Mullerian Hormone” and “Phenotype” or “Phenotypic”. In addition, the reference lists of relevant publications were manually searched to include further relevant studies.

### Study Selection

The reviewers screened independently the titles and abstracts of the retrieved articles based on the predefined inclusion and exclusion criteria. Full-text articles were obtained for potentially relevant studies. Any disagreements between the reviewers were resolved through discussion and consensus.

### Data Extraction

Data extraction was performed independently by 2 reviewers using a standardized data extraction spreadsheet. Any discrepancies were resolved through discussion and consensus. Data collected included study characteristics (authors, publication year, study design, country of the study), participant characteristics—mean age and SD, mean BMI and SD by phenotype; mean AMH levels and SD by phenotype, and AMH assay used. In cases where age and BMI were not reported separately for each PCOS phenotype, the overall mean values for the entire PCOS cohort in the same study were used. When articles reported only the median and interquartile range without providing the SD, a formula was applied to estimate the mean and SD [10]. This approach allowed the inclusion of these studies in the meta-analysis, ensuring consistency in combining results across trials. In the studies where AMH levels were measured in pmol/L, we converted these values to ng/mL using the conversion factor, where 1 ng/mL is equivalent to 7.14 pmol/L [11]. To achieve greater reliability in the results, AMH values were converted to Beckman Coulter Generation II (Gen II) assay standards using validated formulas: Access AMH = 0.128 + 0.781 × Gen II for Access assay, Elecsys AMH = 0.253 + 0.688 × Ansh Labs and Elecsys AMH = 0.087 + 0.729 × Gen II for Ansh and Elecsys assays [11], Gen II = 1.40 × DSL − 0.62 for DSL [12], and Gen II = 1.353 × AMH (IOT) + 0.051 for Immunotech (IOT) [13]. The Elecsys formula was also applied to VIDAS AMH values because of their high correlation [14]. Studies that did not specify the AMH assay used or those without a conversion method were excluded from the final Gen II results. These conversion formulas were used for calculating both the mean AMH and SD, ensuring consistency in measuring proportional variations across different assays.

### Assessment of Risk of Bias

The assessment of the risk of bias for the included studies was conducted using The Risk Of Bias In Non-randomized Studies - of Exposure (ROBINS-E) tool [15], a comprehensive instrument specifically tailored for evaluating bias in observational epidemiological studies, which evaluates seven domains including confounding, selection of participants, classification of interventions, deviations from intended interventions, missing data, measurement of outcomes, and selection of the reported result. Two independent reviewers applied the ROBINS-E tool to each study, with any discrepancies resolved through discussion or consultation with a third reviewer.

### Data Synthesis

The statistical analysis was executed using the R software environment, incorporating the “meta”, “metaphor”, and “ggplot2” libraries for meta-analysis and data visualization. We opted for a random-effects model because of the expected study-to-study variability. The extent of heterogeneity was quantified using the *I*^2^ statistic, which measures the percentage of variation across studies attributable to heterogeneity rather than chance. Additionally, we computed a 95% prediction interval to illustrate where the true effects of similar future studies are predicted to reside. We conducted a sensitivity analysis excluding all studies judged at high risk of bias and those with a total PCOS sample size <100.

To further investigate sources of heterogeneity, we performed a meta-regression using inverse-variance weighted random-effects models. The outcome variable was the mean AMH level per phenotype in each study. Predictor variables included PCOS phenotype (A, B, C, or D), mean age, BMI, and geographic region (Europe, Asia, or Middle East), with phenotype A and Europe used as reference categories. Analyses were conducted for both raw AMH values and values normalized to the Beckman Coulter Generation II assay. Multiple comparisons were adjusted using Tukey's honestly significant difference (HSD) test.

## Results

### Study Selection

**Figure 1.**
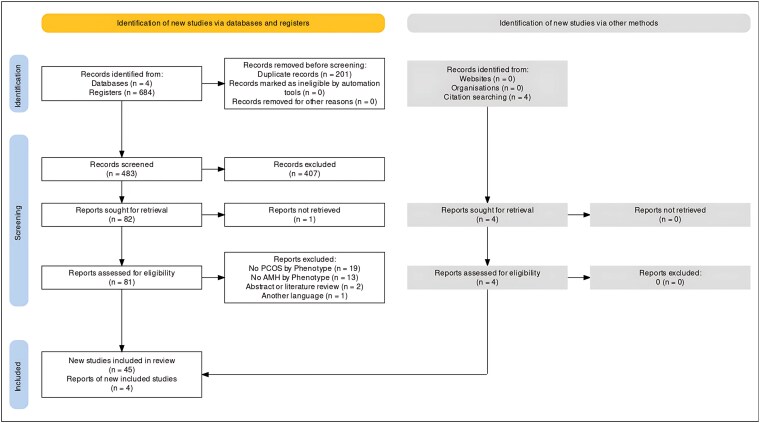
PRISMA flow diagram. Overview of study identification, screening, eligibility, and inclusion for the systematic review and meta-analysis.

The literature search across all four databases yielded a total of 684 citations; of these, 201 were duplicates and were removed. The titles and abstracts of the remaining 483 citations were reviewed for relevance and screened against the inclusion and exclusion criteria. Ultimately, 82 full-text articles were assessed for eligibility. Of these, 35 were excluded, with the most common reason being the lack of specific data on AMH levels by the phenotypes of PCOS. In the end, 49 articles were included in this review, with 4 of them identified through citation searches. Further details on the search strategy and study selection process are presented in the PRISMA flow diagram (Fig. 1).

### Study Characteristics

The systematic review included a total of 49 studies, reflecting a variety of populations. The studies included were conducted between 2009 and 2024, with the number of PCOS women ranging from 31 to 3775, amounting to a total of 15 535 subjects. The geographical distribution of these studies covered a comprehensive range of countries across all continents except Central and South America. The inclusion criteria for age varied among studies, with some specifying a range of 18 to 40 years, whereas others accepted a broader age group. Each study's approach to the inclusion of infertile women also varied, with some studies explicitly including only non-infertile women, whereas others were inclusive or did not specify the fertility status of participants.

In terms of assay methods used to measure AMH levels, a variety of assays were employed. Several studies used the Gen II assay, whereas others used the Roche Elecsys, ACCESS, Immunotech (IOT), DSL, and VIDAS. In some studies, the AMH assay was not determined. This variety in AMH assay methodologies show the importance of considering assay-specific differences when interpreting AMH levels across different populations.

Study characteristics are shown in Table 1.

**Table 1:** Characteristics of included studies

Study	Year	Country	N total PCOS	N PCOS A	N PCOS B	N PCOS C	N PCOS D	AMH assay	AMH PCOS-A (ng/mL)	AMH PCOS-B (ng/mL)	AMH PCOS-C (ng/mL)	AMH PCOS-D (ng/mL)
Adamska 2020	2020	Poland	141	67	30	28	16	BC	9.96	9.33	9.1	9.03
Albahlol 2023	2023	Egypt	185	88	N/A	41	56	ACCESS	9.44	N/A	8.58	11.16
Alebić 2015	2015	Croatia	272	92	N/A	62	118	BC GEN II	7.42	N/A	4.69	6.68
Amini 2018	2018	Iran	635	372	28	34	201	N/A	8.66	06.08	5.70	9.22
Armstrong 2022	2022	USA	227	76	48	50	53	ACCESS	9.95	6.83	6.43	06.03
Bahadur 2021	2021	India	307	114	25	18	150	ELISA	10.51	2.60	3.60	5.34
Bell 2021	2021	Australia	31	7	10	12	2	ELISA ANSH	12.77	4.53	10.30	12.36
Bhide 2017	2017	UK	192	91	N/A	42	59	BC GEN II	12.17	N/A	9.51	10.39
Bozdag 2019	2019	Turkey	78	20	4	36	18	ELECSYS	5.64	2.20	3.20	3.10
Cao NT 2018	2019	Vietnam	479	79	15	61	324	ELECSYS	9.81	9.11	8.65	8.74
Carmina 2016	2016	USA/Italy	35	N/A	N/A	20	15	BC GEN II	N/A	N/A	5.5	5.4
Carmina 2022	2022	USA	274	149	24	94	7	BC GEN II	10.2	5.2	6	5.3
Cela 2018	2018	Italy	44	7	4	9	24	N/A	10.57	4.00	7.78	8.29
De Vos 2018	2018	Belgium	295	66	N/A	54	175	IMMUNOTECH	10.26	N/A	7.4	8.3
Eftekhar 2019	2019	Iran	351	127	56	66	102	N/A	10.72	7.21	8.93	9.36
Fraissinet 2017	2017	France	639	346	2	114	177	BC GEN II	10.08	3.59	7.43	7.72
Gupta 2019	2019	India	57	57	N/A	N/A	N/A	BC GEN II	12.02	N/A	N/A	N/A
Halder 2023	2023	India	131	87	23	8	13	ELISA	10.57	9.45	8.67	10.95
Huijgen 2015	2015	Netherlands	218	94	9	9	106	BC GEN II	N/A	N/A	N/A	7.86
Hwang 2013	2013	Korea	164	59	N/A	N/A	105	IMMUNOTECH	10.1	N/A	N/A	10.0
Ibrahim 2022	2022	India	200	50	50	50	50	N/A	9.43	7.13	8.53	9.71
Jamil 2016	2016	Iraq	263	139	10	36	78	BC GEN II	5.62	03.01	5.53	5.25
Le NSV 2021	2021	Vietnam	119	6	2	16	95	ELECSYS	7.35	2.73	7.94	7.35
Mackens 2020	2020	Belgium	320	140	N/A	20	160	ELECSYS	12.4	N/A	7.7	10.4
Mahajan 2019	2019	India	133	64	4	35	30	ELECSYS	09.05	3.32	6.31	6.39
Malhotra 2023	2023	India	608	273	31	74	230	ELISA ANSH	12.28	10.52	12.45	11.42
Mitra 2023	2023	India	144	91	6	5	42	N/A	11.66	06.08	8.89	12.28
Ozay 2020	2020	Turkey	350	117	89	72	72	CUSABIO ELISA	9.17	8.15	7.30	6.18
Piouka 2009	2009	Greece	100	25	25	25	25	DSL	9.2	6.3	5.1	5.3
Rachmawati 2023	2023	Indonesia	43	17	12	7	7	N/A	10.31	4.18	07.02	7.51
Ramezanali 2016	2016	Iran	386	168	103	83	32	BC GEN II	6.8	5.1	6.3	5.8
Romualdi 2016	2016	Italy	117	73	10	13	21	BC GEN II	9.27	04.05	5.87	7.62
Sachdeva 2019	2019	India	164	111	18	29	6	ELECSYS	11.34	8.69	9.44	7.76
Sahmay 2013	2013	Turkey	251	119	26	45	61	DSL	9.50	03.06	6.12	8.2
Sahmay 2018	2018	Turkey	286	204	16	51	15	DSL	8.34	2.43	6.61	9.28
Santhiya 2021	2021	India	60	9	4	19	28	VIDAS	7.5	7.4	5.8	5.6
Si M 2023	2023	China	1313	596	53	135	529	N/A	9.25	6.88	8.96	7.16
Song 2017	2017	Korea	207	120	87	N/A	N/A	BC	17.7	9.7	N/A	N/A
Sova 2019	2019	Finland	230	106	N/A	N/A	124	VIDAS	12.84	N/A	N/A	8.54
Sumji 2023	2023	India	113	41	17	21	34	ELISA ANSH	12.67	7.28	8.91	11.87
Svetlana 2019	2019	Russia	100	53	27	15	5	BC	10.97	4.66	5.20	9.56
Tian 2014	2014	China	160	40	40	40	40	DSL	8.33	4.29	5.49	6.70
Urbanovych 2020	2020	Ukraine	80	18	19	20	23	ELECSYS	11.1	6.1	4.1	10.1
Vural 2023	2023	Turkey	154	54	35	44	21	ELECSYS	7.8	5.7	5.8	5.5
Wiweko 2014	2014	Indonesia	71	21	2	3	45	BC GEN II	11.1	11.5	8.72	8.2
Wiweko 2018	2018	Indonesia	125	39	27	26	33	BC GEN II	13.33	11.33	11.96	6.6
Yue CY 2018	2018	China	653	189	175	163	126	UNION	9.9	8.9	9.3	9.1
Zhang 2023	2023	China	3775	2430	183	N/A	1162	ELECSYS	9.23	4.43	N/A	6.99
Zhao 2023	2023	China	220	84	34	N/A	102	N/A	8.89	9.81	N/A	6.60

Summary of study year, country, sample sizes for total and PCOS phenotypes (A, B, C, D), AMH levels (ng/mL), and assay methods.

Abbreviations: BC, Beckman Coulter; ACCESS, Access Immunoassay; BC GEN II, Beckman Coulter Generation II; ELECSYS, Roche Elecsys; IMMUNOTECH, Immunotech; DSL: Diagnostic Systems Laboratories; N/A, not available; UNION, Union Immunoassay); VIDAS, bioMérieux VIDAS [17, 19, 20, 31-75].

### Risk of Bias of Included Studies

The risk of bias within the included studies was assessed using the ROBINS-E tool. Most studies were judged at low risk in domains related to measurement of AMH and outcomes, as well as in reporting, reflecting methodological robustness in these aspects. However, relevant concerns emerged in other domains. Bias due to participant selection was frequent, as many studies were conducted in tertiary infertility clinics, potentially overrepresenting women with more severe phenotypes and limiting generalizability. Bias due to confounding was also common, reflecting the retrospective design of many studies and incomplete adjustment for factors such as age, BMI, and metabolic status. In addition, several studies relied on a single AMH measurement without longitudinal follow-up, which may have introduced measurement bias. Overall, most studies were classified as moderate risk of bias, with a smaller subset at high risk. These limitations emphasize the need for cautious interpretation of the results and may partly explain the heterogeneity observed across studies.

**Figure 2.**
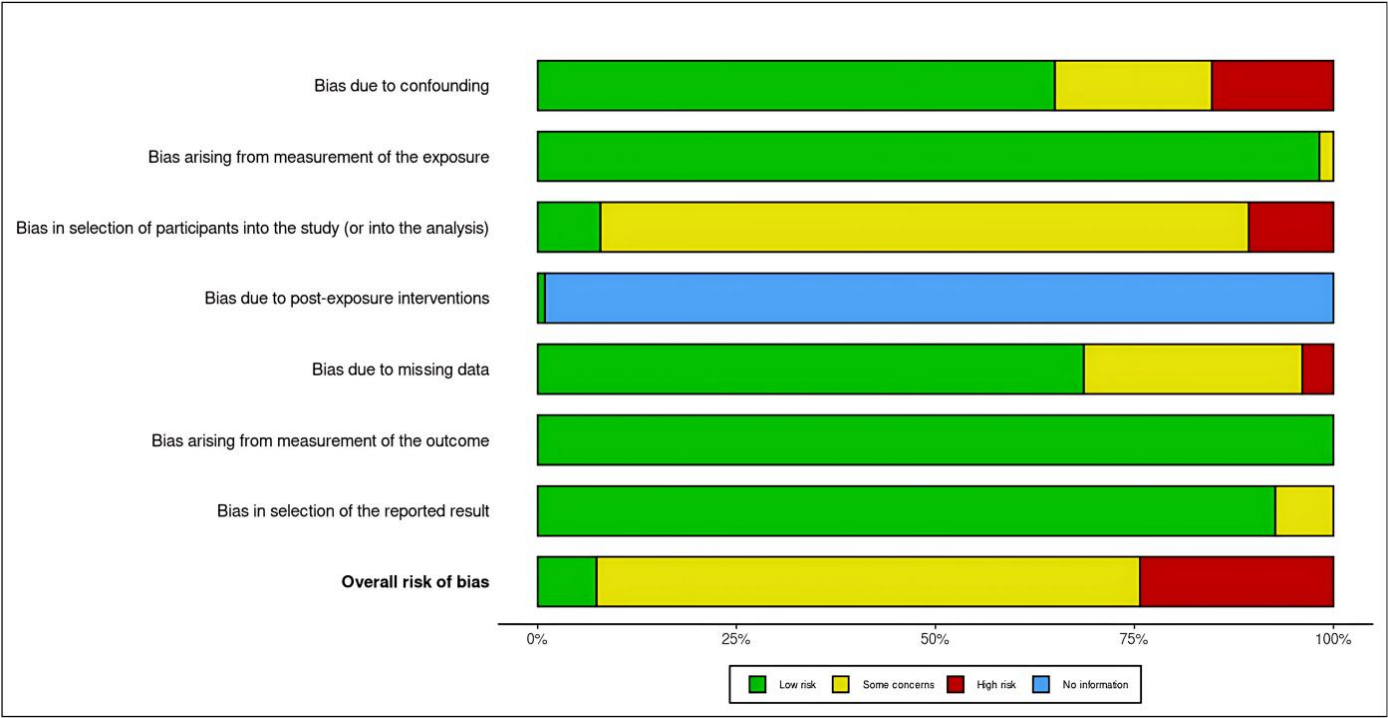
Risk of bias assessment using ROBINS-E. Graphical summary of the risk of bias evaluation for the included studies. The figure shows the proportion of studies categorized as low risk, some concerns, high risk, and no information across the specified domains. Detailed study-level assessments are provided in the Supplementary Material [16].

The summary of this risk assessment is presented in Fig. 2, whereas detailed results for each individual study are provided in the Supplementary Material [16].

### Synthesis of Results

**Figure 3.**
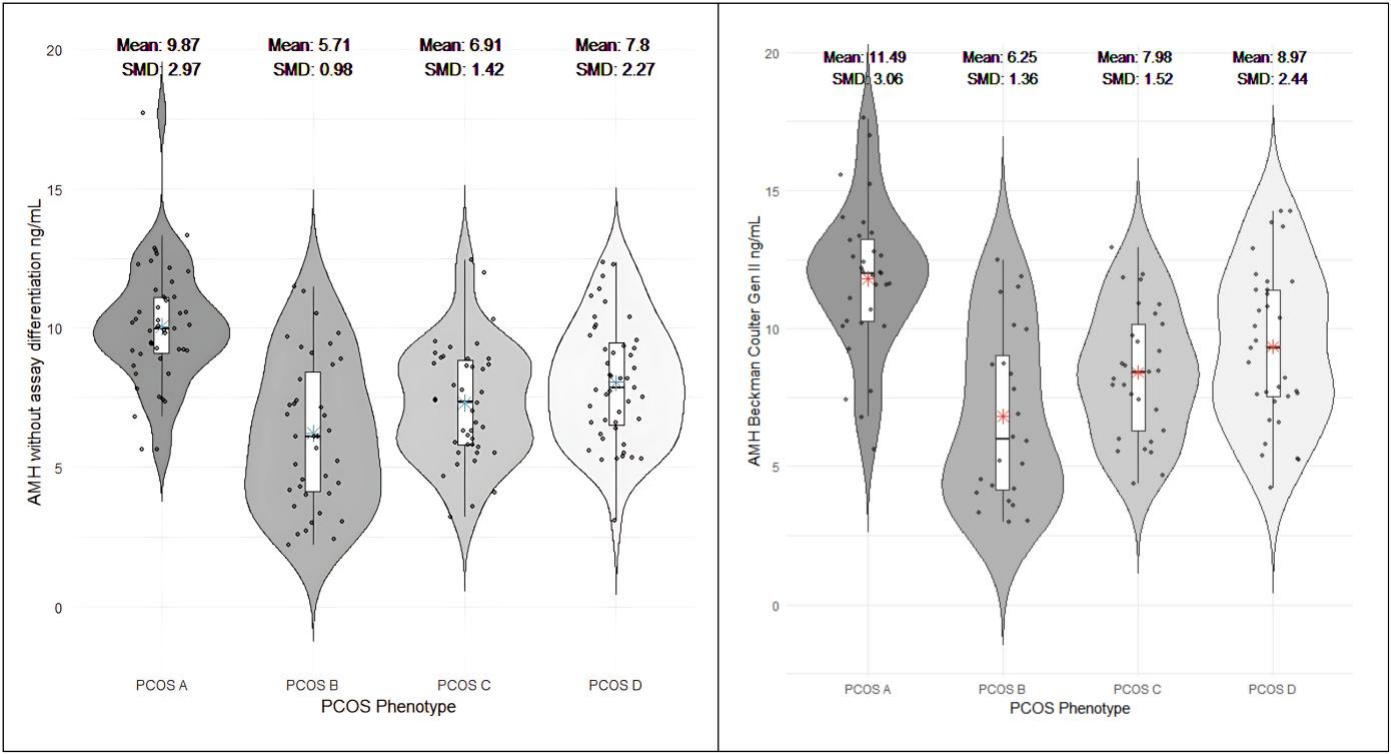
AMH levels across PCOS phenotypes. Violin plots displaying the distribution of AMH levels (ng/mL) across PCOS phenotypes A, B, C, and D, without assay differentiation (left) and after Beckman Coulter Gen II assay normalization (right). Mean values and standardized mean differences (SMD) are highlighted for each phenotype.

The results from our systematic review highlight significant variability in AMH levels when stratified by PCOS phenotypes (Fig. 3). The PCOS phenotype A had the highest mean AMH level measured without assay differentiation at 9.87 ng/mL (standardized mean difference [SMD] 2.97; 95% CI, 9.29-10.48), and with the Beckman Coulter Gen II assay conversion at 11.49 ng/mL (SMD 3.06; 95% CI, 10.47-12.61). In contrast, phenotype B showed the lowest median AMH levels in both measurements, 5.71 ng/mL (SMD 0.98; 95% CI, 4.96-6.57) without assay differentiation and 6.25 ng/mL (SMD 1.36; 95% CI, 5.20-7.51) with Beckman Coulter Gen II. PCOS C mean AMH without assay differentiation 6.91 ng/mL (SMD 1.42; 95% CI, 6.30-7.57) and converted to BC GEN II 7.98 ng/mL (SMD 1.52; 95% CI, 7.16-8.90), PCOS D without assay differentiation 7.80 ng/mL (SMD 2.27; 95% CI, 7.20-8.45), and BC GEN II 8.97 ng/mL (SMD 2.44; 95% CI, 8.04-10.00).

In sensitivity analyses excluding all high-risk studies and those with total PCOS was N < 100. After applying these criteria, 28 studies remained for analysis. The pooled estimates and the AMH hierarchy remained essentially unchanged (A > D > C > B). In the overall dataset, mean AMH levels were: A, 9.84 ng/mL (95% CI, 9.30-10.40); B, 6.35 (5.40-7.46); C, 7.19 (6.44-8.03); and D, 8.09 (7.35-8.90). When restricted to studies standardized to the Beckman Coulter Gen II assay, the pattern persisted: A, 11.71 (10.40-13.20); B, 6.71 (5.40-8.33); C, 7.95 (6.76-9.34); and D 9.38 (8.23-10.69). Supplementary Figures display the corresponding forest plots [16].

**Figure 4.**
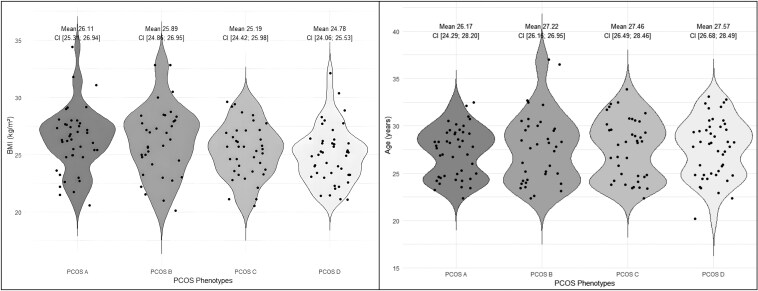
Age and BMI distribution across PCOS phenotypes. Violin plots illustrating the distribution of age (years) and body mass index (BMI, kg/m^2^) among PCOS phenotypes A, B, C, and D. Mean values with CIs are displayed for each phenotype.

The age distribution across phenotypes was relatively similar, with means ranging from 26.17 to 27.57 years, indicating that age may not be a distinguishing factor in AMH levels among the included studies. BMI also presented low differences, with the mean BMI being the lowest in phenotype D at 24.78 kg/m^2^ and high in PCOS A (mean 26.11 kg/m^2^) (Fig. 4).

Our results showed notable disparities in sample sizes across the 4 PCOS phenotypes, reflecting the prevalence of each phenotype in the studied populations. Phenotype A displayed the largest sample sizes, suggesting that this phenotype is more commonly studied and also more prevalent. Larger sample sizes generally provide more precise estimates, as indicated by the narrower CIs in our forest plots. This implies that our findings for phenotype A might be the most reliable indicator of AMH levels among the PCOS phenotypes we studied.

**Figure 5.**
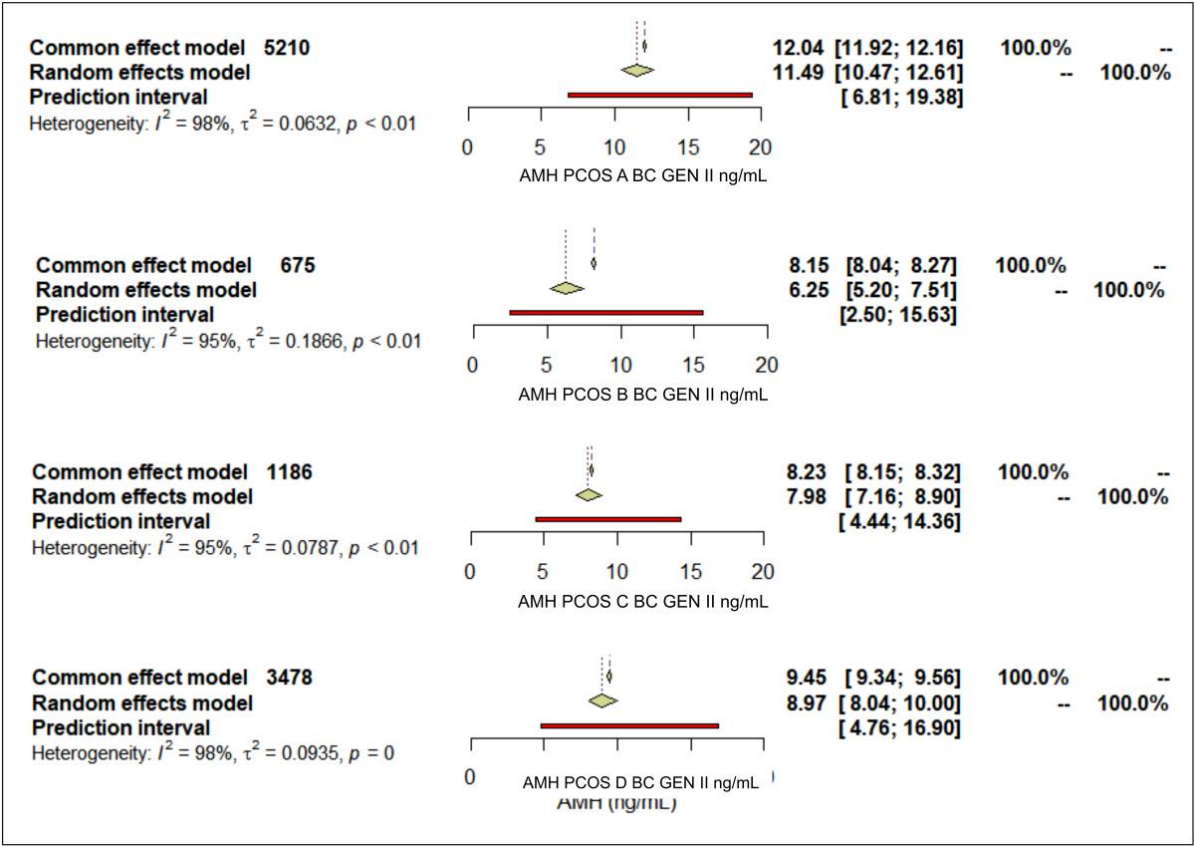
Forest plot of AMH levels by PCOS phenotypes without assay differentiation. Forest plot illustrating mean AMH levels (ng/mL) and 95% CIs for PCOS phenotypes A, B, C, and D. Results were calculated using random-effects models.

In contrast, phenotype B often had smaller sample sizes, indicating that this phenotype might be less common, less frequently studied, or more challenging to diagnose. As a result, although the estimated AMH levels for phenotype B are valuable, they are subject to more variability and might require cautious interpretation and verification in larger cohorts. Phenotypes C and D had moderate sample sizes, providing a balance between the extremes of A and B (Fig. 5).

Additional forest plots displaying subgroup analyses and details of the meta-analysis are provided in the supplementary material [16].

### Meta-regression Analysis

**Figure 6.**
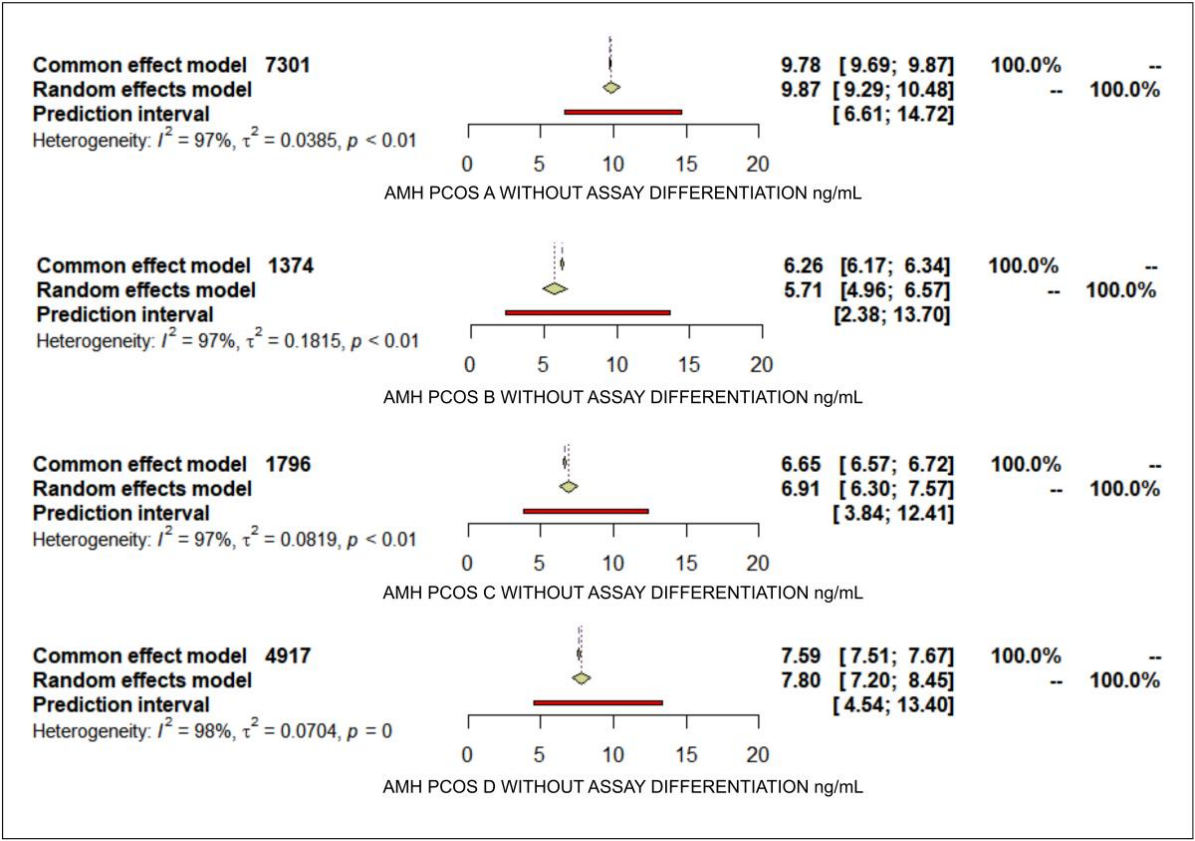
Forest plot of AMH levels by PCOS phenotypes converted to Beckman Coulter Gen II assay. Forest plot showing mean AMH levels (ng/mL) and 95% CIs for PCOS phenotypes A, B, C, and D, standardized to Beckman Coulter Gen II assay values.

To better understand sources of heterogeneity in AMH levels across PCOS phenotypes, we performed a meta-regression using a random-effects model weighted by inverse variance. The dependent variable was the mean AMH level for each phenotype within each study. Independent variables included PCOS phenotype (A, B, C, or D), mean age, mean BMI, and study region (Europe, Asia, Middle East), using phenotype A and Europe as reference categories. Analyses were conducted for both raw AMH values and those normalized to Beckman Coulter Gen II assay standards (Fig. 6).

**Figure 7.**
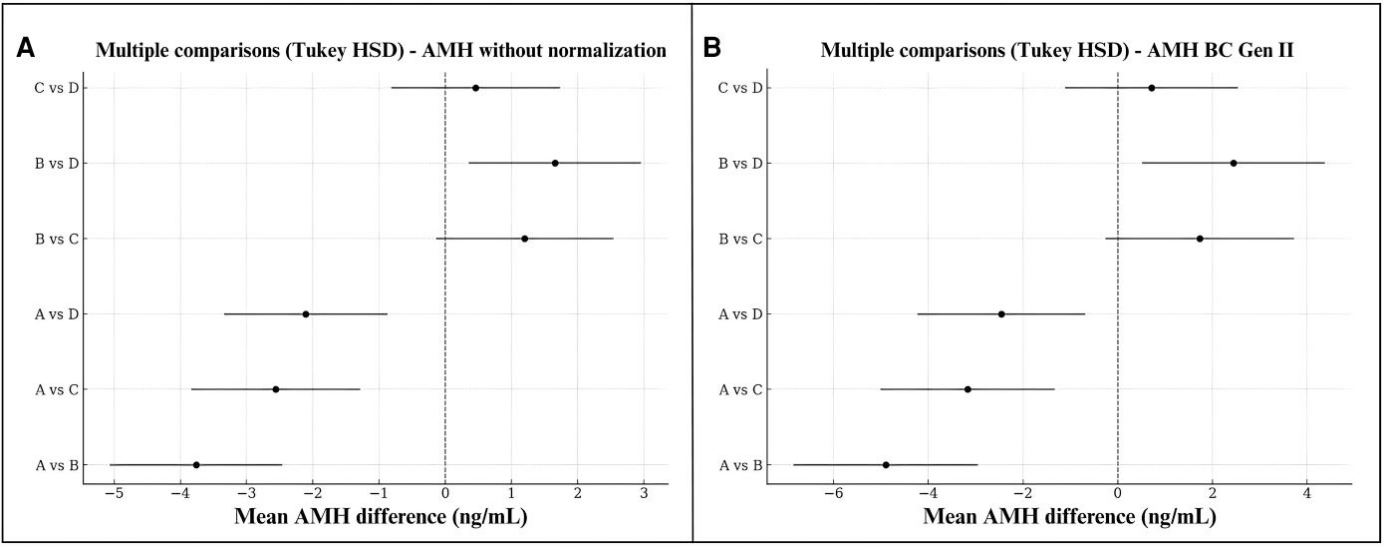
Multiple comparisons of mean AMH levels between PCOS phenotypes by Tukey HSD test. A = based on unnormalized values, whereas B = results with data normalized to the Beckman Coulter Generation II assay.

Compared to phenotype A, all other phenotypes (B, C, and D) had significantly lower AMH levels (*P* < .001 in all models), with consistent direction and magnitude of effect across both raw and normalized data. Tukey HSD tests confirmed statistically significant differences between phenotype A and all others in both models. In the normalized model, additional significant differences were observed between phenotypes B and C (*P* = .038) and between B and D (*P* < .001), whereas C vs D showed a borderline result (*P* = .074). These results reinforce a hierarchy of AMH levels across phenotypes: A > D ≈ C > B (Fig. 7).

Among continuous covariates, BMI showed a negative association with AMH in the unadjusted model (−0.09 ng/mL per 1-kg/m^2^ increase; 95% CI, −0.16 to −0.007; *P* = .033), but this association lost statistical significance in the normalized model, likely because of reduced assay-related variability. Age was not significantly associated with AMH levels in either model, possibly reflecting the relatively similar age distribution among study participants.

Regional analysis revealed significantly lower AMH levels in studies from the Middle East compared to Europe (−5.17 ng/mL; 95% CI, −7.33 to −3.00; *P* < .001 in the normalized model). Region accounted for approximately 40% of the between-study heterogeneity (*R*^2^ = 0.405).

## Discussion

### Principal Findings

Although recent studies have investigated AMH levels in women with PCOS, few have stratified AMH concentration by Rotterdam phenotypes using standardized conversion formulas to Gen II assay values. This study contributes uniquely by offering a phenotype-specific analysis across 49 studies, correcting for assay variability. Our meta-analysis found significant variability in AMH levels across different phenotypes of PCOS. Phenotype A consistently exhibited the highest AMH levels, followed by phenotypes D, C, and B. These results align with previous research indicating that women with more severe ovulatory dysfunction or hyperandrogenism, particularly in phenotype A, tend to have elevated AMH levels [17, 18]. Interestingly, we observed that phenotype D had higher AMH levels than phenotype C, suggesting that oligoanovulation may exert a greater influence on AMH levels than hyperandrogenism alone. This is supported by studies showing that ovulatory dysfunction is closely linked to follicular excess, which in turn elevates AMH levels [19]. Conversely, phenotype B showed the lowest AMH levels, this supports the hypothesis that polycystic ovarian morphology is the primary factor driving elevated AMH levels in PCOS, as previously suggested by Carmina et al. [20], who found that PCOM correlates more strongly with AMH than hyperandrogenism or oligoanovulation.

These findings were confirmed and refined through meta-regression analysis. After adjustment for age, BMI, and geographic region, phenotypes B, C, and D maintained significantly lower AMH levels compared to phenotype A (*P* < .001 for all comparisons), reinforcing the robustness of the observed hierarchy: A > D ≈ C > B. The BMI showed a modest inverse association with AMH in the raw data model but lost significance after assay normalization, whereas age showed no association, likely because of the relatively narrow age distribution across included studies. Additionally, region of origin explained approximately 40% of the between-study heterogeneity, with studies from the Middle East reporting significantly lower AMH levels than those from Europe (*P* < .001). This pattern aligns with reports showing that, in women from Saudi Arabia and African countries, AMH cutoff values below 4.0 ng/mL are diagnostic for PCOS, whereas higher thresholds (4.2-5.6 ng/mL) are more predictive in European populations and even higher values (4.4-10 ng/mL) in Asian cohorts [21]. Such discrepancies may reflect methodological differences, regional variation in phenotype distribution, assay heterogeneity, or population-specific characteristics.

The distinct pattern of AMH levels across PCOS phenotypes underscores its potential clinical value for phenotype-specific approaches. Recent evidence indicates that higher AMH levels are associated with more adverse metabolic and endocrine profiles, particularly in phenotype A, and may therefore guide counseling on long-term cardiometabolic risks [22].

Longitudinal evidence from the CARDIA study demonstrated that women meeting National Institutes of Health criteria of PCOS (mainly Rotterdam phenotypes A and B) had increased risks of diabetes, dyslipidemia, subclinical atherosclerosis, and greater left ventricular mass compared with women without PCOS [23].

Although phenotypes A and B are both more hyperandrogenic and insulin resistant than phenotypes C and D, women with the classic phenotype defined by hyperandrogenism and anovulation exhibit greater impairment in insulin-mediated substrate utilization [24]. Compared with other phenotypes, they diverge markedly in AMH levels, with phenotype A displaying the highest and phenotype B the lowest concentrations, this highlights that AMH reflects an ovarian-driven dimension of PCOS distinct from metabolic features.

Moreover, AMH has been shown to predict the response to ovulation induction: in women with PCOS-related infertility, each 1-ng/mL increase in baseline AMH reduced the odds of ovulation by approximately 10%, and women with AMH >8 ng/mL were significantly less likely to respond to clomiphene or clomiphene plus metformin [25]. These findings suggest that AMH may complement phenotype differentiation and serve as an adjunct for tailoring treatment strategies.

### Comparison with Existing Literature

Previous meta-analyses, such as those by Anand et al [26] and Tsukui et al [27], have focused on the diagnostic accuracy of AMH in distinguishing PCOS patients from controls, consistently showing elevated AMH levels in women with PCOS. A recent study showed the potential AMH as a diagnostic biomarker for PCOS. A serum AMH concentration of at least 5.39 ng/mL was associated with an increased risk of PCOS, with a sensitivity of 88.6%, specificity of 92.75%, an area under the curve of 0.95 [28]. However, these analyses did not differentiate between the various PCOS phenotypes, which we believe is crucial given the metabolic heterogeneity within the syndrome. As highlighted by Soares Jr et al (2023) [29], different PCOS phenotypes exhibit distinct metabolic profiles, with phenotype A, in particular, being associated with a higher risk of metabolic syndrome and type 2 diabetes. This reinforces the importance of stratifying PCOS by phenotypes to better understand the variability in clinical and metabolic outcomes. Our study adds to the literature by providing insights into the variability of AMH levels across these phenotypes, suggesting that PCOM plays a more significant role in elevating AMH levels than other features, such as hyperandrogenism or ovulatory dysfunction.

### Strengths and Limitations

A major strength of this meta-analysis is the inclusion of a large sample size (15 535 women) from diverse populations, which enhances the applicability of our findings to broader clinical contexts. Additionally, the use of conversion formulas for standardizing AMH measurements across different assays adds reliability to the results. However, the meta-analysis revealed a pronounced degree of heterogeneity in AMH levels, as evidenced by *I*^2^ values close to or at 98% across all phenotypes. This substantial variability suggests that factors beyond phenotypic classification, such as differences in study populations, ethnicity, assay methodologies, and inclusion criteria, may influence AMH concentrations. Small-study effects, methodological discrepancies, and potential publication bias might also contribute to this heterogeneity. These findings underscore the complexity of PCOS as a syndrome with a wide clinical spectrum and highlight the limitations of using AMH as a uniform diagnostic biomarker.

One important contributor to this variability is assay heterogeneity. We normalized AMH values to the Gen II assay, which has historically been used as a reference due to its greater analytical sensitivity at higher concentrations [30]. As expected, normalization increased mean AMH levels across phenotypes and improved their differentiation. Nevertheless, heterogeneity remained high (*I*^2^ > 95%), indicating that assay standardization alone cannot fully account for between-study differences. More recent automated assays, such as Elecsys (Roche Diagnostics) and Access (Beckman Coulter), have been widely adopted in clinical practice but consistently yield lower concentrations compared with Gen II (approximately 0.86-0.88 times lower) [13, 14]. These discrepancies highlight the urgent need for international reference standards and validated conversion formulas before AMH can be reliably applied as a phenotype-specific clinical tool in PCOS.

Furthermore, the variability in sample sizes across phenotypes, particularly the smaller cohort for phenotype B, may affect the precision of estimates for this subgroup and warrants cautious interpretation. In addition, because the studies did not include control groups, it was not possible to explore normative AMH ranges, which could have provided additional clinical context. Future studies including both PCOS phenotypes and healthy controls will be important to refine population-specific thresholds.

### Conclusions and Implications

Our findings suggest that AMH levels could serve as an important diagnostic indicator for differentiating between PCOS phenotypes, particularly when integrated with other clinical parameters [20]. The distinct AMH profiles across phenotypes underscore the need for phenotype-specific approaches in PCOS management. However, the variability in AMH assays across studies highlights the importance of standardized measurement techniques to ensure consistent clinical application [31]. Moving forward, future research should focus on reducing heterogeneity by standardizing AMH measurements and exploring the role of AMH in guiding personalized treatments for PCOS.

## Data Availability

The data underlying this article are available within the article and its online supplementary material. Additional data will be shared upon reasonable request to the corresponding author.

## Clinical Trial Information

**Registration number:** PROSPERO Registration ID: CRD42023444193

## Abbreviations

AMH: anti-Müllerian hormone
BMI: body mass index
HA: hyperandrogenism
HSD: Tukey's honestly significant difference
IOT: Immunotech
PCOM: polycystic ovarian morphology
PCOS: polycystic ovary syndrome
ROBINS-E: The Risk Of Bias In Non-randomized Studies - of Exposure
SMD: standardized mean differences
